# The ACP Evidence-Based Guide to Complementary and Alternative Medicine

**DOI:** 10.1155/2011/601271

**Published:** 2010-09-22

**Authors:** Sungmi Lian

**Affiliations:** Department of Family Medicine, The University of Texas Medical Branch, 301 University Boulevard, Galveston, TX 77555-1123, USA

## Abstract

Complementary and alternative medicine (CAM) is no longer new terminology in the healthcare system but, *evidence-based CAM* is still an unfamiliar term. Evidence-based medicine, a practice of medicine based on the recommendation derived from a systematic, scientific study of published data, is accepted as the standard in the healthcare. *ACP Evidence-Based Guide to Complementary and Alternative Medicine* by Bradly Jacobs and Katherine Gundling is reviewed. Up-to-date reference books like the *ACP Evidence-Based Guide to Complementary and Alternative Medicine* is an essential tool for improving quality of care when the providers aim to practice evidence-based medicine.

Complementary and alternative medicine (CAM) is no longer new terminology in the healthcare system but, *evidence-based CAM *is still an unfamiliar term. Evidence-based medicine, a practice of medicine based on the recommendation derived from a systematic, scientific study of published data, is accepted as the standard in the healthcare system since the introduction of the term in a publication in 1992 in the Journal of the *Journal of the American Medical Association.* Drs. Bradly Jacobs and Katherine Gundling have successfully organized the most recent data and presented it in a reader-friendly format in the *ACP Evidence-Based Guide to Complementary and Alternative Medicine*. 

There are several features in this book which makes it a valuable reference to use in a primary care office. First, each chapter is dedicated to discussing broad aspects of the most commonly seen disorders in primary care. I found book chapters listed by disorders much more useful than chapters listed by different therapies or modalities. This is because both patients and providers look for possible CAM therapies for certain conditions or symptoms, not vice versa. Most commonly used products or therapies are discussed in details within the chapter for an in-depth understanding of the treatment protocol. Chapter 3, which is dedicated to allergic disorders, includes a detailed section on different types of CAM diagnostic tests for allergic disorders, and whether each has proven effective or not based on research data. Different types of allergy testing have been gaining popularity among patients. I have been asked often by patients to perform those tests or to make a clinical diagnosis based on the results they have received from other CAM providers. This knowledge allows providers to educate and counsel patients to make an informed decision.

Next, I like that the authors used a progressive approach in evaluating the quality of evidence by adapting a grading system called Grading of Recommendations Assessment, Development and Evaluation (GRADE). Some of the advantages of GRADE over other systems include clear and explicit definitions of each grade and systematic consideration of the study designs, quality, and consistency to name a few. This system is endorsed by many reputable international organizations including the World Health Organization, UpToDate, and American College of Physicians. End-of-chapter summary chart and recommendations for each type of therapy is invaluable at providing time-saving yet up to date information. 

Yet, another reason for why this book is a valuable resource is the safety information the treatment. As a clinician, the level of safety of the treatment options is just as or even more important than the efficacy. Level of safety is evaluated and rated from *double thumbs up* to *double thumbs down.*

Finally, I considered the highlight of this book to be, the end of each chapter evidence summary of CAM treatment table which summarized the discussion in each of the chapters. The summary table includes clinical indication, type of therapy, dosage regimen if known, efficacy (grade A to D), and safety profile along with clinical recommendations. This summary table can be effectively used as a quick reference to look for the effectiveness and safety of a product or therapy for a certain condition, or to search for effective possible treatment options for the conditions. I also found it to be a useful reference while teaching medical students and residents in a clinical setting.

One reservation that I have for this book is the section on cancer which could have been expanded to include more information regarding evaluation of specific treatment options, herbs, and supplements, and beneficial or harmful effects in different types of cancer. Prevalence of cancer increases every year as well as the CAM use by cancer patients, and current evidence-based guideline of CAM in oncology will be highly valuable for providers and patients. 

Our patients have high expectations of healthcare providers and actively seek advice regarding CAM. They also seek advice for effective and safe CAM for their conditions as well. Healthcare providers fundamental knowledge of CAM by is important for providing high-quality care for patients. Up-to-date reference books like the *ACP Evidence-Based Guide to Complementary and Alternative Medicine* is an essential tool for improving quality of care when the providers aim to practice evidence-based medicine. This book would be especially beneficial for those with no structured training in CAM. I strongly recommend this book to all healthcare providers and hope that they find this book informative and enjoyable as I did.

